# Case report: A prosthetic valve endocarditis caused by *Legionella bozemanae* in an immunocompetent patient

**DOI:** 10.3389/fmed.2022.1055465

**Published:** 2022-11-03

**Authors:** Mai Sasaki Aanensen Fraz, Gry Dahle, Kirsten Margrete Skaug, Sophie Jarraud, Stephan Frye, Jørgen Vildershøj Bjørnholt, Ingvild Nordøy

**Affiliations:** ^1^Department of Microbiology, Oslo University Hospital, Oslo, Norway; ^2^Centre for Rare Disorders, Oslo University Hospital, Oslo, Norway; ^3^Department of Medicine, Lovisenberg Diaconal Hospital, Oslo, Norway; ^4^Department of Cardiothoracic Surgery, Oslo University Hospital, Oslo, Norway; ^5^National Reference Centre for Legionella, Institute of Infectious Agents, Hospices Civils de Lyon, Lyon, France; ^6^Faculty of Medicine, Institute of Clinical Medicine, University of Oslo, Oslo, Norway; ^7^Section for Clinical Immunology and Infectious Diseases, Oslo University Hospital, Oslo, Norway; ^8^Research Institute of Internal Medicine, Oslo University Hospital, Oslo, Norway

**Keywords:** endocarditis, prosthetic valve endocarditis (PVE), molecular diagnostic techniques, blood culture negative endocarditis, *Legionella bozemanae*, *Legionella bozemanii*

## Abstract

Extrapulmonary infections with *Legionella species* are rare, but important to acknowledge. We report a case of infective endocarditis (IE) with *Legionella bozemanae* in a 66-year-old immunocompetent man with an aortic homograft. The diagnosis was made by direct 16S rRNA gene amplification from valve material, confirmed by a targeted *Legionella*-PCR in serum and the detection of *L. bozemanae* specific antibodies. To our knowledge, this is the first confirmed case of IE with *L. bozemanae* as causative pathogen. The infected aortic prosthesis was replaced by a homograft, and the patient was successfully treated with levofloxacin and azithromycin for 6 weeks.

## Introduction

The *Legionellaceae* are widespread in water and soil environment, and can colonize manmade water sources. This is also true for *Legionella bozemanae*, which has also been found in commercial potting soils (1–4). *L. bozemanae* is uncommonly a pathogen, but it has been reported to be able to cause severe and necrotizing pneumonia in immunocompromised patients (5, 6). Extrapulmonary infections with *L. bozemanae* are even rarer, however cases of arthritis and soft tissue infections are described (7–9). There are 18 previous reports of infective endocarditis (IE) caused by *Legionella species* in the English literature, and in only two of these, the affected valve was native (10–20). The presentation of *Legionella* endocarditis tends to be subacute, with weight loss, low-grade fever and anemia, and without peripheral manifestations as embolic events or immune complex depositions (21). Exceptions from this are two reports of bacterial seeding to the central nervous system and one case of digital microembolisms (15, 19, 20). In the previous reports of *Legionella* endocarditis, the majority of IE patients had no preceding or concurrent airway infection, with only four of 18 cases reporting clinical or radiological findings consistent with pneumonia. In one case series, the *Legionella* endocarditis cases were believed to be part of hospital outbreaks of *Legionella* (10). Another feature of the reported *Legionella* IEs in the literature is that although small valvular vegetations are often visible at surgery, they are frequently undetectable by echocardiograms (10, 17). Accordingly, as the patients also often display only moderate signs of inflammation, the diagnosis of IE can be delayed.

## Case description

**FIGURE 1:**
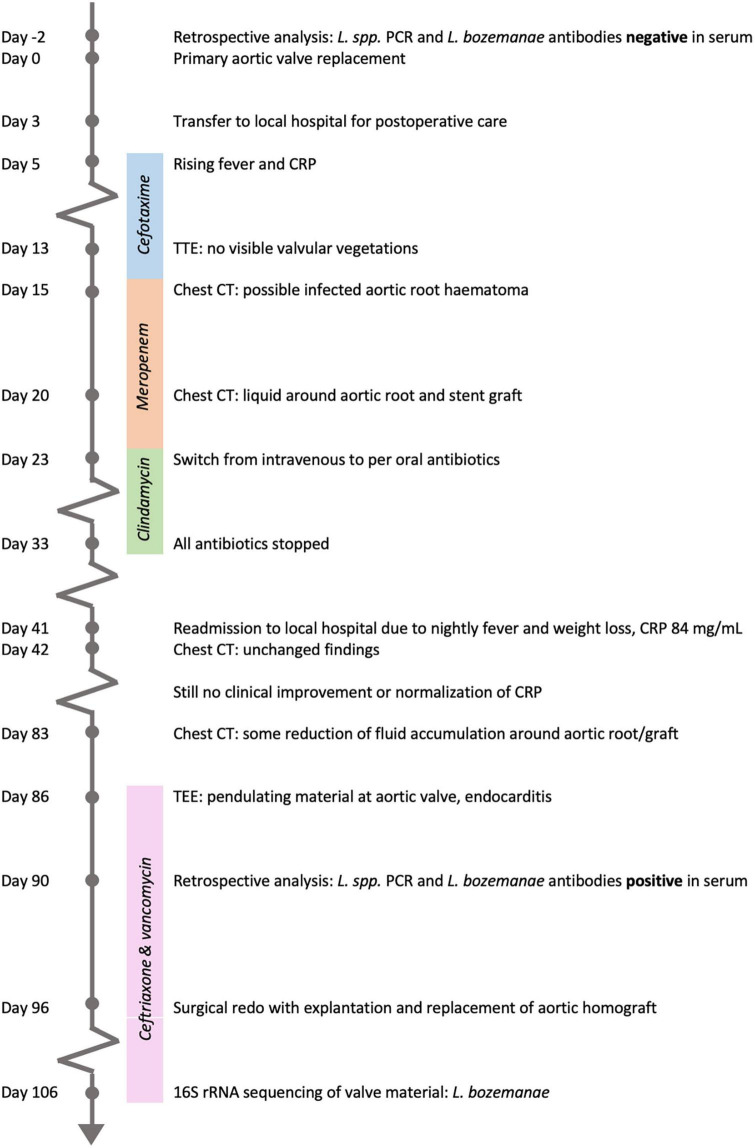
Timeline from admission for the primary aortic valve replacement until microbial diagnosis by 16S rRNA sequencing of valve material.

A 66-year-old immunocompetent man with an increasingly symptomatic aortic stenosis and bicuspid aortic valve underwent aortic valve replacement in April 2021 at Oslo University Hospital, Rikshospitalet. He was otherwise healthy, besides an occasional migraine. The inserted prosthetic aortic valve was a biological 23 mm Perimount prosthesis (Edwards Lifesciences, Irvine, CA, United States), and an aorta Intergard woven graft 30 mm (Getinge, Göteborg, Sweden) was also inserted (Day 0). The surgical procedure was uneventful, and per operative echocardiogram and postoperative chest x-ray were normal. The further disease course, diagnostics and treatment in this case is illustrated in the timeline of Figure 1.

The patient was transferred to his local hospital for postoperative care on Day 3 after surgery. Physical examination at the day of transfer revealed no heart murmur or pulmonary crepitations. During the first postoperative week he developed fever and CRP increased to a maximum of 329 mg/L on Day 8. Antibiotic treatment with cefotaxime was started. There were no visible valvular vegetations by transthoracic echocardiogram on Day 14. As his condition failed to improve, a chest CT was performed on Day 15 displaying a possible infected aortic root hematoma. CT also demonstrated pleural- and pericardial fluid, but no lung parenchymal infiltrations. In this period, the patient complained of a non-productive troublesome cough. CRP levels did not fall significantly, and the antibiotic treatment was switched to meropenem. Pleural fluid was drained. Subsequently, CRP decreased to 34 mg/L and the patient’s condition somewhat improved. The patient was discharged to his home with a 10 days course of oral clindamycin.

**FIGURE 2:**
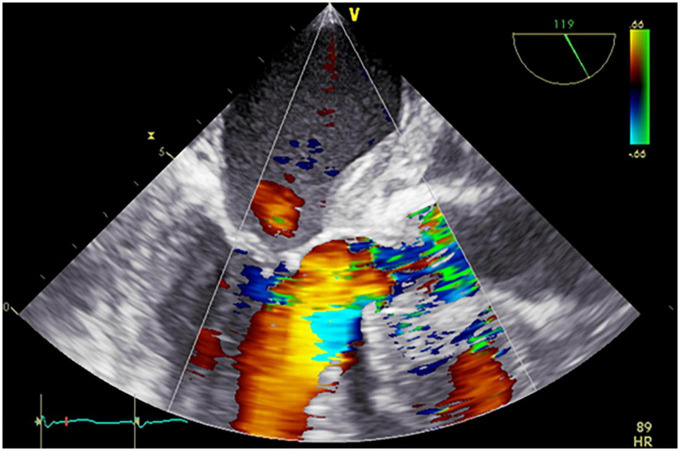
Pre-operative transesophageal echocardiogram. Severe aortic regurgitation and thickened aortic wall.

However, he continued to have nightly fever and weight loss after the discharge. He also had a short self-limiting episode of pain, rubor and swelling in his left knee. On Day 41 he was readmitted to his local hospital with increasing fever, and CRP had increased to 84 mg/L. There were normal findings by physical examination of his knee joint. Chest CT was repeated on Day 42 with unchanged findings around the aortic root, and still no lung parenchymal infiltrations. The patient was observed without starting any antibiotic treatment, with the aim of catching a pathogen by repeated blood culturing. He stayed afebrile the following 2 days, and was again discharged without any blood culture findings. However, the patient did not improve clinically, and CRP was continuously moderately elevated. Therefore, chest CT was again repeated on Day 83, this time revealing some reduction of fluid accumulation around the aortic root and ascendens graft. Finally, transesophageal echocardiogram was performed Day 86, showing pendulating material at the ventricular side of the aortic valve cusps, consistent with endocarditis (Figure 2). The patient had at this time developed a second degree atrioventricular block, and a temporary pacemaker was inserted.

**FIGURE 3:**
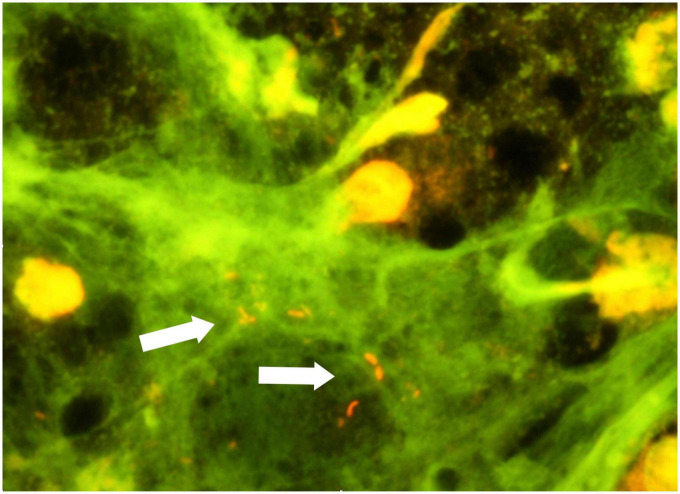
Excised valve material, acridin stain. Rods marked by white arrows.

As blood cultures remained negative, the patient was treated empirically with ceftriaxone and vancomycin from Day 86. The patient’s condition deteriorated despite this treatment, and he was decided to undergo redo surgery. The Perimount prosthesis and the ascendens graft were excised and replaced by a Homograft 22 mm (Cell and Tissue laboratory, Sahlgrenska, Sweden) on Day 96. Microscopy of acridin stained excised valve material demonstrated scarcely distributed rods (Figure 3), but culture of the explant on standard bacterial and fungal media did not yield growth. However, direct 16S rRNA gene amplification of valve material yielded sequences of 720 base pairs matching 100% with *L. bozemanae* in the National Center for Biotechnology Information (NCBI) Blast database and EzBioCloud (22). The valve tissue DNA extraction and 16S rRNA gene amplification were performed twice with same result. Seeding of the explanted valve material was also done on buffered charcoal-yeast extract (BCYE) media for *Legionella*, but we were not able to grow *L. bozemanae.* The patient was finally treated with levofloxacin and azithromycin for 6 weeks. The atrioventricular block progressed to a total block after the redo, and a permanent pacemaker was implanted after 4 weeks. Transthoracal echocardiogram was performed by the end of the antibiotic treatment, displaying an aortic graft in satisfactory position, without leakage. Post treatment CT arteriography showed significant decline of fluid around the aortic root. The patient is now physically fit and is doing well, a year after completed therapy.

An environmental search was performed to detect *Legionella spp.* in water sources at the local hospital, at the university hospital where the surgery was performed and at the patient’s home. *L. bozemanae* was not detected in any of these locations.

The diagnosis of *L. bozemanae* was confirmed retrospectively by a hybridization probe-based dual-color LightCycler real-time PCR with primers that amplify a 378-bp product within the 16S rRNA gene of *Legionella spp.* of the patient’s serum (23). The serum was sampled and stored frozen from 6 days (Day 90) before the reoperation. Subsequent melting point analysis corresponded with *L. bozemanae.* Additionally, an assay to detect *L. bozemanae* specific antibodies was performed by the French National Reference Center for Legionella (Lyon, France). Serum from 2 days before the primary operation (Day-2), was negative (<1:16) for *L. bozemanae* antibodies, while in serum from Day 90 the titer was positive (>1:2048).

Finally, although the diagnosis was certain, we performed 16S Nanopore sequencing on both cardiac valve material and serum from Day 90 to see if this method would detect *L. bozemanae.* DNA was extracted by SelectNAplus from Molzym (Bremen, Germany) and prepared for Nanopore sequencing using a 16S Barcoding Kit (SQK-RAB204, Oxford Nanopore Technologies, Oxford, UK). Sequencing was performed on a MinION flowcell (FLO-MIN106D, Oxford Nanopore Technologies, Oxford, UK) for 43 h. Sequences were filtered by size and quality using ProwlerTrimmer and blasted against the NCBI 16S rRNA refseq database, which was complemented with a full-length sequence of the 16S rRNA gene of *L. bozemanae*, extracted from the whole genome assembly GCF_900640135.1. While the serum sample from Day 90 yielded 36,673 reads before filtering and 10,678 after, the valve sample only gave 153 reads with 90 reads left after filtering. No *Legionella* specific sequences were found in the serum-derived reads. On the other hand, 89 of the 90 (98%) reads from the valve sample were identified as *L. bozemanae* specific.

## Discussion

Infective endocarditis has a high mortality rate, and early identification of the causative microbe is important to target antibiotic therapy and improve outcome. *Legionella spp.* are not routinely recoverable from blood cultures and will not be covered by standard empiric antibiotic treatment for endocarditis. A subacute disease course without typical IE findings adds to making the diagnosis of this infection challenging. This case report has several take-away lessons: Most important, although *Legionella spp.* are infrequent causes of infectious endocarditis in general, they should be considered in culture negative cases of post-surgery prosthetic valve endocarditis (PVE) (24). This case also demonstrates that PVE caused by *L. bozemanae* is possible in an otherwise healthy and immunocompetent host.

Furthermore, this and most other similar cases show that the *Legionella* bacteria’s way of entrance to cause endocarditis often remains unknown. Although *Legionella* is often associated with airway infection, the literature shows that airway symptoms are often not present in *Legionella* PVE. Our patient experienced a protracted disease course after the primary surgery, with fluctuating fever and CRP. Repeated chest CT showed no pulmonary infiltrations, and *Legionella* was not detected by *Legionella* PCR in bronchial lavage fluid after the reoperation. The patient experienced some dry cough, but this could as well be attributed to pleural irritation by pleural fluid present. *L. bozemanae* was neither detected in the water systems of the operating hospital, the local hospital nor the patient’s house.

When it comes to treatment, there are no randomized trials involving legionellosis with non-pneumophila species. For long, antimicrobial susceptibility testing (AST) for *Legionella* has been challenging and not standardized, until the European Society of Clinical Microbiology and Infectious Diseases (ESCMID) Study Group for Legionella Infections recently issued a recommendation (25). In this case, AST was impossible due to lack of a positive culture. The literature regarding antibiotic susceptibility for *Legionella species* other than pneumophila is scarce, but AST studies performed by broth microdilution and in a human monocyte cell line demonstrate higher activity of quinolones than erythromycin against *L. bozemanae* (26). However, fluoroqinolone resistance may be induced during antibiotic treatment (27). Based on this knowledge and the gravity of the infection in this case, our patient was given a combination of levofloxacin and azithromycin. However, long QT interval should always be considered and monitored when combining these drugs (28).

We identified the microbial cause of endocarditis by broad-range 16S rRNA gene amplification of the excised specimen. This is common practice in blood culture negative endocarditis (BCNE) when explanted valve material is available, and can identify a microbial cause in about two thirds of cases (29). In cases of IE where surgery has not been performed, a reliable molecular method to detect relevant microbial genetic material in blood, serum or plasma would be an ideal diagnostic tool. Unfortunately, as of today no such method has shown satisfactory performance. Metagenomic next-generation sequencing of plasma is suggested as a promising aid in systemic infections, although both false positive and false negative results, as well as the noise of polymicrobial identification can make interpretation of results challenging (30–32). Our 16S Nanopore sequencing of the patient’s serum were not able to detect any *Legionella*.

In this case, targeted *Legionella* PCR was positive in serum from 6 days before the redo. The European Society of Cardiology guidelines recommend the use of targeted PCR of EDTA-blood when IE with *Bartonella spp*., *Tropheryma whipplei* or fungi are suspected (33). The guidelines also recommend systematic serological testing for *Legionella pneumophila* in BCNE according to local epidemiology. However, most serological assays for *Legionella* only detect *L. pneumophila*, which leaves a diagnostic gap for other *Legionella* spp. that may be underdiagnosed. Serological assays for *Legionella species* other than pneumophila are not avaliable in Norway, and is in general not widely performed as there are no commercial tests. International laboratory collaboration partners or networks can be helpful in cases like ours. Targeted *Legionella* PCR is on the other hand more commonly performed, and is mostly used in airway specimens. The sensitivity of targeted *Legionella* PCR in sera of patients with *Legionella* pneumonia has previously been reported to be 30–63% depending on the target gene, and the specificity to be 100% (34–36).

In conclusion, *Legionella* endocarditis is difficult to diagnose due to both indistinct clinical findings and the fact that microbiological identification depends on the clinicians’ suspicion. Targeted *Legionella* PCR of serum should be considered in cases of culture negative PVE, and *Legionella* spp. as a cause of BCNE are likely underdiagnosed.

## Data availability statement

The genomic sequences presented in this case report are included in the Supplementary material. Further inquiries can be directed to the corresponding author.

## Ethics statement

Written informed consent was obtained from the individual(s) for the publication of any potentially identifiable images or data included in this article.

## Author contributions

MF collected the data and drafted the manuscript. KS, SF, SJ, and JB analyzed and interpreted the data. GD and IN provided clinical care for the patient. All authors reviewed, edited, and approved the final manuscript.
