# Supplementary material for: Case report: A prosthetic valve endocarditis caused by Legionella bozemanae in an immunocompetent patient
Source: Front Med (Lausanne). 2022 Nov 3;9:1055465. doi: 10.3389/fmed.2022.1055465 (PMC9669447; doi:10.3389/fmed.2022.1055465)
Supplement: Supplementary file 2 [file Table_2.DOCX]

LightCycler real-time PCR, 378-bp Legionella bozemanae:

GTTGATAGGTTAAGAGCTAATTAACTGGACGTTACCCACAGAAGAAGCACCGGCTAACTCCGTGCCAGCAGCCGCGGTAATACGGAGGGTGCGAGCGTTAATCGGAATTACTGGGCGTAAAGCGTGCGTAGGTGGTTGATTAAGTTATCTGTGAAATCCCTGGGCTTAACCTGGGCAGGTCAGATGATACTGGTTGACTCGAGTATGGGAGAGGGTAGTGGAATTTCCGGTGTAGCGGTGAAATGCGTAGAGATCGGAAGGAACACCAGTGGCGAAGGCGGCTACCTGGCCTAATACTGACACTGAGGCACGAAAGCGTGGGGAGCAAACAGGATTAGATACCCTGGTAGTCCACGCTGTAAACGATGTCAACTAGCT
